# Retraction: Dual-phenotype hepatocellular carcinoma: correlation of MRI features with other primary hepatocellular carcinoma and differential diagnosis

**DOI:** 10.3389/fonc.2024.1502292

**Published:** 2024-10-17

**Authors:** Frontiers Editorial Office

The journal retracts the 2023 article cited above.

Following publication, concerns were raised about the duplicate publication of the article cited above in Frontiers in Oncology and Journal of Digital Imaging:

Gu, H. X., Huang, X. S., Xu, J. X., Zhu, P., Xu, J. F., & Fan, S. F. (2023). Diagnostic Value of MRI Features in Dual-phenotype Hepatocellular Carcinoma: A Preliminary Study. Journal of Digital Imaging, 36(6), 2554-2566.

An investigation was conducted in accordance with Frontiers’ policies, whereby it was confirmed that data was shared between both groups of authors, who submitted manuscripts for consideration in two journals simultaneously.

Following the Committee on Publication Ethics guidelines, this article, published most recently is retracted.

This retraction was approved by the Chief Executive Editor of Frontiers. The authors agree to this retraction.

Frontiers would like to thank the concerned reader who contacted us regarding the published article.

